# A systematic review of burnout among doctors in China: a cultural perspective

**DOI:** 10.1186/s12930-018-0040-3

**Published:** 2018-02-08

**Authors:** Dana Lo, Florence Wu, Mark Chan, Rodney Chu, Donald Li

**Affiliations:** 1 University Health Service, The Hong Kong Polytechnic University, Hong Kong, China; 2 Department of Applied Social Sciences, The Hong Kong Polytechnic University, Hong Kong, China; 3 The Jockey Club School of Public Health and Primary Care, The Chinese University of Hong Kong, Hong Kong, China; 4 WONCA World Executive Council, Hong Kong, China

## Abstract

**Background:**

Numerous studies around the world has already suggested that burnout among doctors is a global phenomenon. However, studies for burnout in doctors are relatively limited in Chinese communities when compared to the West. As risk factors, barriers to intervention and strategies combatting burnout in different parts of the world can vary a lot due to different social culture and healthcare system, study with a focus at doctors in China from a cultural perspective is a worthful endeavor.

**Methods:**

Systematic searches of databases were conducted for papers published in peer-reviewed journals from 2006 to 2016. Selection criteria included practicing doctors in Mainland China and publications written in English or Chinese. Keywords searched including “burnout”, “doctors” and “China” in 3 electronic databases has been undergone. Traditional understanding of “work attitude” and “doctors’ humanity” from ancient Chinese literature has also been retrieved.

**Results:**

Eleven full papers, including 9302 participants, were included in this review. The overall prevalence of burnout symptoms among doctors in China ranged from 66.5 to 87.8%. The review suggested that negative impact of burnout include association with anxiety symptoms and low job satisfaction at the individual doctors’ level, and prone to committing medical mistakes affecting patient safety and higher turnover intention at the society/organizational level. Burnout was higher among doctors who worked over 40 h/week, working in tertiary hospitals, on younger age group within the profession (at age 30-40), and with negative individual perception to work and life.

**Conclusions and implications:**

The overall prevalence and adverse impact of burnout among doctors in China echo with the findings from Western studies. Young doctors and doctors working in tertiary hospitals are more at risk of burnout, probably related to shift of social culture related to the loss of medical humanities and a weak primary healthcare system. Potential strategies of managing burnout in Chinese doctors should therefore take consideration from the Chinese cultural perspective, with renaissance of medical humanities and strengthening the primary healthcare system in China.

## Background

The initial description of burnout first appeared in the mid-1970s in the United States [1]. Most publications for burnout studies since then have targeted on human service workers, with doctors fall into the high risk category of professionals suffering from burnout due to their exposure to high levels of stress in their daily work. Maslach and Jackson [2] well conceptualized burnout as a 3-dimensional construct consisting of emotional exhaustion, depersonalization, and reduced personal accomplishment. The Maslach Burnout Inventory (MBI) has been widely used in research studies as a gold standard of measurement. Consequences of burnout have been well studied including adverse outcomes on patient care [3, 4], professionalism [5], doctors’ own health and safety [6, 7] and the viability of healthcare systems, such as reductions in doctor’s professional work effort [8, 9].

In a recent survey of US physicians found that 54.4% of the respondents reported at least one symptom of burnout in 2014 [10]. An earlier survey from rural British Columbia showed that 80% of physicians suffered from moderate-to-severe emotional exhaustion, 61% suffered from moderate-to-severe depersonalization, and 44% moderate-to-low feelings of personal accomplishment [11]. A burnout prevalence rate of 28.9% was reported amongst the responding cohort of UK Oto-rhino-laryngologists [12]. A cross-sectional study on burnout among French GPs in training reported 16.0% with high levels of emotional exhaustion, 33.8% with high score for depersonalization, and 38.9% had high score for low personal accomplishment [13]. A study on burnout in Australian specialist anaesthetists reported that 20% with high emotional exhaustion, 20% with high levels of depersonalization and 36% with low levels of personal achievement respectively among the respondents [14]. The overall prevalence of high personal burnout was 50% in New Zealand’s senior doctors and dentists, with many attributing their feelings of burnout to work conditions [15].

Studies for burnout in doctors were relatively limited in Asian regions when compared to the West. In a cross-sectional study in Yemen, 63.2% of respondent doctors showed high emotional exhaustion, 19.4% showed high depersonalization, and 33.0% showed low personal accomplishment [16]. The reported burnout rate among doctors in Malaysia indicated a rate of 36.6% [17]. A cross-sectional survey on burnout among public doctors in Hong Kong reported 31.4% of the respondents satisfied the criteria for high burnout [18]. The overall burnout rate of doctors in China was reported range from 66.5% [19] to 76.9% [20] with high burnout ranged from 12.1% [21] to 25.4% [22]. As the number of studies is limited and most of them are cross-sectional studies performed in Asia, simple direct comparison of burnout condition is difficult. The alarming figures quoted in the available studies may suggest an underestimated problem below the tip of the iceberg.

Numerous studies over the world has already suggested that burnout among doctors is a global phenomenon. Estimate of burnout in doctors often yields high figures and varies among countries, across time, specialties or sector of work, i.e. public/private or rural/urban [23]. Hence, an in-depth research and discussion on burnout management should take reference at the specific doctors’ group working, in similar locality and setting, with consideration to their own values and culture.

Regarding to China, having an enormous population base of 1.4 billion (equivalent to 19% of the world population) [24], doctors in China experience long working hours with tremendous workload. Healthcare has been one of the important themes of deepening reform, mentioned in China’s National People’s Congress in 2014 [25], as deterioration of the doctor-patient relationship has become a huge problem in China’s healthcare system, with one-third of doctors experienced conflict and thousands have been injured [26].

### Literature review on traditional Chinese culture on working

*“Culture is a vague and elusive concept most individuals seldom explain or consciously think about and evaluate, and yet it is imperative.”* [27]. *Confucianism* is the leading culture and social value for traditional Chinese. It was developed from the teachings of the Chinese philosopher *Confucius* (551–479 BC) who considered himself a re-transmitter of the values of the *Zhou* dynasty, a golden age in ancient China. Harmony is the core value in Confucian Chinese culture. To maintain harmony, the way of governing the society and families by *Confucianism* is to have good compliance to rites (*Li*), a system of ritual norms and propriety that determines how a person should properly act in everyday life, and is one of the basic Confucian ethical concepts and practices. In a society governed by *Li*, every person is expected to behave according to the *Li* assigned to his respective social role or status. A good compliance to *Li* can maintain the “face” of an individual. Hu [28], a renowned anthropologist in Chinese culture, stresses that “face” is not only limited to reputation achieved through success and ostentation, such as wealth, which is recognized externally, but also includes the respect of the group for a man with good moral reputation. It is both a social sanction for enforcing moral standards and an internalized sanction. If an individual is not compliant to *Li*, the behaviour of this individual is considered as incorrect and inappropriate. This individual loses “face” and will then lead to a perception of shame. Shame in Chinese also carries two levels of meaning, namely the external perception which requires the presence of others, and the internal awareness by self, which does not require the presence of others [29]. Hence, *“… the superior man must be watchful over himself when he is alone”* i.e. good compliance to *Li*, even when not being observed by others, which is well documented in the chapter of *Da Xue*, *Book of Rites (Liji)* [30]. Otherwise, the individual will have a self-awareness of shame. This is a form of internalized sanction.

The Chinese attitude of studying and working has also been well documented in the *Book of Rites*, stating that “*…the scholar learns extensively, but never allows his researches to come to an end; he does what he does with all his might, but is never weary.”* The attitude of working or the sense of mission of fulfilling the role of one’s duty is reflected clearly from the *Later Memorial for the case to go to war (Post Chu Shi Biao*), submitted by *Zhuge Liang*, chancellor-regent of the Shu Han in the Three Kingdoms period (220-280 AD), to Shu’s second emperor. The most famous and historic quote from this report is: *“I strive to do my best until I die.”*. One possible deduction regarding to work attitude from Chinese is, to use all one’s effort to complete one’s duty without complaining even when no one is observing, is perceived as an appropriate behaviour with high moral value, i.e. good compliance to *Li* and preservation of “face”. On the contrary, if an individual complains or shows any sign of unable to cope while fulfilling one’s assigned duty, he may be viewed as incompetent, and be perceived as ‘inappropriate behaviour’ with assigned low moral value, i.e. non-compliance to *Li* and loss of “face”. To the best understanding, the concept of work-life balance has not been well illustrated in historic and contemporary Chinese culture.

To the best understanding, this is the first systematic review of cross-sectional surveys on the current burnout condition of doctors in China. No other review has provided an overview of the influence of the traditional Chinese understanding of “work” and “doctors’ integrity” with reference to doctor’s burnout. With different social culture and healthcare system, risk factors, barriers to prevention or intervention, the future strategies of tackling burnout in different parts of the world should also vary; this is also the first review paper discussing the acceptance of burnout by Chinese doctors from a traditional Chinese cultural perspective, including the potential impact of Confucianism, shift of social culture and diminishing trend of humanity in medicine in China. Discussion on the strategic management of burnout is based on the observation of cultural shift and conflicting values among Chinese young doctors, the major workforce in the coming decades for the healthcare system in China.

This study reviews current knowledge on burnout among doctors in China, addressing the following three research questions:

- What do we know about the prevalence of burnout among doctors in China?
- What is the adverse impact or conditions associated with burnout in China?
- What are the predictive factors of burnout among doctors in China, with specific reference to the traditional Chinese understanding of doctor’s role?

## Methods

A comprehensive search in January 2017 was conducted by searching keywords including “burnout”, “doctors” and “China” in 3 electronic databases: PubMed, Medline, and PsycINFO. In addition, manual review on the reference list of included papers has been performed as well. The search strategy for the literature search is summarized in Table 1.

**Table 1:** Search criteria

Prevalence terms	
	AND
Factor terms	
Filters	Published between January 2006 and December 2016 Practising doctors English or Chinese languages Working Experience

Titles of the abstracts were screened for appropriateness by the first author. The first and second author further reviewed the abstracts with close reference to the inclusion and exclusion criteria (presented in Table 2) to eliminate studies not meeting the selection criteria. Full texts of criteria-met articles were retrieved. Reference lists of the identified papers were scanned through and searched to maximize the search.

**Table 2:** Selection criteria

Criteria	Included	Excluded
Publication type	Published in peer-reviewed scientific journals Reporting original research results Ancient Chinese literature referring to doctor’s integrity and concept of work Written in English or Chinese	Book chapters, editorials, dissertations, theses and conference abstracts “Grey” literature (for example, policy advocacies, media or journal comments)
Study design	Cross-sectional study design Quantitative studies providing doctor-specific burnout data using burnout measures with validity support from commonly accepted sources of evidence	Qualitative studies Studies not providing burnout related data/measurement
Population	Practising doctors Practice in Mainland China Age	Non-doctors (medical students, and non-doctor health care providers such as nurses and traditional Chinese medicine practitioners were excluded) Practice not within China (Hong Kong SAR, Macau SAR and Taiwan were excluded due to different medical systems)

Studies were considered methodologically strong or weak based on:

- *Design* Longitudinal and prospective studies were considered as methodologically stronger than cross-sectional and exploratory studies.
- *Representativeness* Studies with populations of > 100 respondents, studies with response rates > 75% and multisite studies were considered as stronger.
- *Instruments* Studies that used valid and reliable questionnaires (α > 0.60) were considered stronger than studies that used questionnaires for which validity and reliability were not sufficient or not reported; likewise, studies that reported on cut-off scores were considered stronger.
- *Statistics* Studies that used more advanced statistical analyses were considered as stronger than those that used descriptive statistics only.

Relevant information from ancient Chinese literature was also retrieved, enabling a deeper understanding on the social expectation of being a doctor in the traditional Chinese culture and the role of humanities in medicine.

## Results

**Fig. 1:**
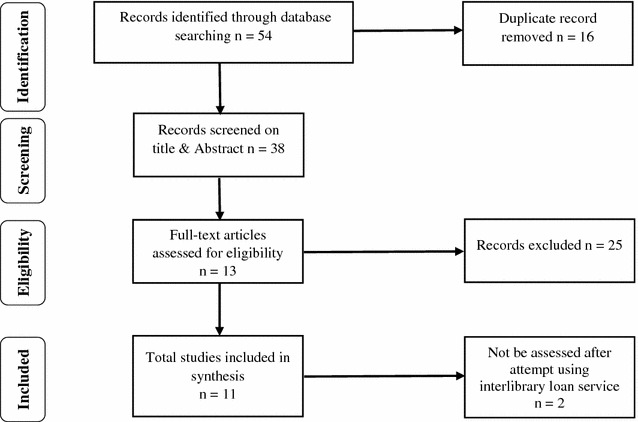
Flow diagram of article selection

Regarding the search of doctors’ burnout with reference to peer-reviewed journals, the search process resulted in a total of 54 articles respectively by 3 electronic databases: PubMed (32), Medline (13) and PsycINFO (9). As the total number of articles was manageable by initial search, year of publication and language limitation were not applied at the preliminary search. After elimination of duplicates, the search result was 38. The titles and abstracts of the studies were reviewed again by considering the inclusion and exclusion criteria. The flow diagram of article selection is presented in Fig. 1.

There were 13 abstracts meeting the criteria. However, two papers could not be assessed after attempt using interlibrary loan service. As a result, eleven full papers were included in this review, with majority (8 out of 11) published within 5 years. All concerned papers were published in English with the exception that two were written in Chinese. The characteristics of the searched studies are presented in Tables 3 and 4.

**Table 3:** Characteristics of included studies

Author/year	Study name	Design/data collection wave and year	Population/characteristics	Regions	No. of centers	Training status/specialty	Measuring instrument	Findings
Zhou 2016 [34]		Cross-sectional survey	n = 1274	Heilongjiang	2	Trained Multi but not specified	15-item CMBI	Score 1–7
Wen 2016 [20]		Cross-sectional survey	n = 1537	Sichuan, Chongqing, Gansu, Guizhou, Guangdong, Shanxi, Hunan, Zhejiang, Yunnan, Ningxia	46	Not stated Multi but not specified	15-item CMBI-GS	Weighed sum score equation
Jin 2015 [39]		Cross-sectional survey	n = 135	Shanghai	2	Intern Not stated	15-item CMBI-GS	Score 1–7
Xiao 2014 [22]		Cross-sectional survey	n = 205	Beijing	3	Not stated Emergency	15-item CMBI-GS	6 pt
Wang 2014 [19]		Cross-sectional survey	n = 457	Shanghai	21	From junior to senior Multi	19-item CMBI-HSS	Score 1–7
Wu 2013 [21]		Cross-sectional survey	n = 1618	Liaoning	7	From junior to senior Not stated	16-item CBMI-GS	Score 0–6
Cui 2013 [33]		Cross-sectional survey	n = 510	Beijing, Xian, Shanghai, Jiangsu, Zhejiang, Guangdong, Fujian, Hubei, Hunan, Inner Mongolia	≥ 10	From junior to senior Multi	16-item CMBI-GS	Score 0–6
Wang 2012 [38]		Cross-sectional survey	n = 1011	Liaoning	6	From junior to senior Not stated	15-item CMBI-GS	Score 0–6
Zhang 2011 [35]		Cross-sectional survey	n = 1451	Hubei	67	From junior to senior Multi	15-item CMBI	Score 1–5
Wu 2008 [37]		Cross-sectional survey	n = 543	Henan	3	Not stated Multi	16-item CMBI-GS	Score 0–6
Zhu 2006 [36]		Cross-sectional survey	n = 561	Not stated	3	From junior to senior Multi but not specified	16-item CMBI-GS	Score 1–7

**Table 4:** Summary of findings of the included studies

Author/year	Prevalence of burnout	Adverse impact of burnout		Predictive factors of burnout			
		Individual	Society	Workload	Work setting	Sociodemographic	Individual perception
Zhou 2016 [34]		✓					
Wen 2016 [20]	76.9% (some or serious)		✓	✓	✓		
Jin 2015 [39]							✓
Xiao 2014 [22]	25.4% (high)	✓					
Wang 2014 [19]	66.5% (mild or severe)			✓	✓	✓	✓
Wu 2013 [21]	12.1% (high burnout)			✓	✓		✓
Cui 2013 [33]	81.8–87.8% (medium or above)				✓	✓	
Wang 2012 [38]						✓	
Zhang 2011 [35]			✓				
Wu 2008 [37]					✓	✓	✓
Zhu 2006 [36]					✓	✓	✓

Regarding the Chinese literature related to work attitude and doctors’ humanity, the Four Books and Five Classics have been reviewed. These are the Chinese classic texts illustrating the core value and belief systems in Confucianism. “Great Learning” and “Analects” of the Four Books and the “Book of Rites” of Five Classics are chosen for the present review. In addition, two texts of traditional Chinese medical books, “On the Absolute Sincerity of Great Physicians” and the preface of the “Medical Book—Pei Yizhong” are included for the present review.

### Findings from the Chinese classic texts: social expectation of being a doctor in the traditional Chinese culture

To understand burnout phenomenon among Chinese doctors, an important underlying concept is the status of doctor in Chinese culture. It is probably superficial if the meaning of status is just limited to the levels of wealth or official ranking in the society. Looking back to the few 1000 years of Chinese history, doctors in historical record with high honour are those considered having high moral value such as *Sun Simiao* (581–682 AD), a famous traditional Chinese medicine doctor of the *Sui* and *Tang* dynasty. He was titled as *“China’s King of Medicine”* for his significant contributions to Chinese medicine and tremendous care to his patients. Apart from this, he is known for the text “*On the Absolute Sincerity of Great Physicians*,” often called “*The Chinese Hippocratic Oath*,” which comes from the first chapter of his very important medical textbook “*Essential Formulas for Emergencies Worth a Thousand Pieces of Gold*”. The following is an excerpt of the text, highlighting the importance of having high moral standard as a good Chinese doctor:

*A Great Physician should not pay attention to status, wealth or age; neither should he question whether the particular person is attractive or unattractive, whether he is an enemy or friend, whether he is a Chinese or a foreigner, or finally, whether he is uneducated or educated. He should meet everyone on equal grounds. He should always act as if he were thinking of his close relatives* [31].

The preface of a medical book written by *Pei Yizhong*, a doctor in *Ming* dynasty stated that: If the talent and morality of a person are not comparable to fairy and Buddha respectively, he should not become a doctor misleading others. Being a doctor is a sacred mission. It is not a technique serving as a tool to gain one’s living [32]. This has been echoed by *Qiu Fazu* (1914–2008), regarded as the “*Father of Modern Chinese Surgeons*”, in one of his famous motto.

### Research question one: what do we know about the prevalence of burnout among doctors in China?

Five studies investigated the prevalence of burnout among doctors in China.

A multi-center cross-sectional survey in 2016 interviewed 1537 doctors working in multi-specialties from 10 provinces in China reported 76.9% suffering from some or serious burnout symptoms [20]. Another cross-sectional survey in 2014 interviewed 205 doctors working in the Emergency unit of three large general hospitals in Beijing reported 25.4% exhibited high levels of career burnout [22]. A cross-sectional survey published in 2014 interviewed 457 doctors working in multi-specialties from 21 hospitals in Shanghai revealed 60.6% were experiencing a mild degree of burnout and 5.9% were experiencing a severe degree of burnout [19]. Another cross-sectional survey in 2013 interviewed 1618 doctors from 7 teaching hospitals in Liaoning province reported the prevalence of high degree burnout was 12.1% [21]. A multi-center cross-sectional survey in 2013 interviewed 510 doctors working in multi-specialties from 10 areas in China reported 84.9% suffering from medium or higher level of emotional exhaustion, 87.8% with moderate degree of depersonalization and 81.8% with at least moderate degree of diminished personal accomplishment [33]. The training status or year of work experience of the respondents ranged from junior to senior doctors in the studies of Wang, Wu and Cui, but it was not specified in the studies of Wen and Xiao. Doctors from multi-specialties were interviewed in the studies of Wen, Wang and Cui, only doctors from the Emergency unit was interviewed in the study of Xiao, and the specialty information was not specified in the study of Wu. Different MBI versions were used in these five studies with some differences in scoring and grading of burnout. The remaining studies did not report on the burnout figures/prevalence.

### Research question two: what is the adverse impact or conditions associated with burnout in China?

Four studies reported on the adverse impact or conditions associated with burnout in China.

*At the individual doctors’ level*, a cross-sectional survey in 2016 interviewed 1274 doctors working from multi-specialties in Heilongjiang showed that there is a strong positive correlation between job burnout and anxiety symptoms in doctors and the prevalence of anxiety symptoms in their study was 31% [34]. The correlation analysis from another cross-sectional survey conducted in the Emergency unit in Beijing showed a negative correlation between job satisfaction and emotional exhaustion and cynicism, and a positive correlation with reduced personal accomplishment. The two subscales (anxiety subscale and depression subscale) of the psychological distress and the three subscales (emotional exhaustion, cynicism and personal accomplishment) of burnout were correlated significantly with intrinsic and extrinsic job satisfaction in the sampled population, but the correlations between personal accomplishment and extrinsic job satisfaction were not significant. The study concluded that burnout and job satisfaction among Emergency doctors are at a moderate level, and burnout is negatively associated with higher job satisfaction [22].

*At the organizational/society level*, a multi-center study from 10 provinces of China revealed that doctors who reported serious burnout were independently associated with higher incidence of medical mistakes over the course of the last year, ranged from incomplete or incorrect items in patients’ records, to medication errors and delayed treatments [20]. Another cross-sectional survey interviewed 1451 doctors working in multi-specialties from urban state-owned medical institutions in Hebei studied the relationship between job satisfaction, burnout and turnover intention in 2011, showed turnover intention, which significantly and negatively related to all job satisfaction subscales, positively related to each subscale of burnout syndrome. The study concluded that job satisfaction had both significant direct effects and indirect effects through occupational burnout as a mediator on turnover intention [35].

### Research question three: what are the predictive factors of burnout among doctors in China?

All studies attempt to find out the risk and/or protective factors of burnout.

*Regarding to the workload factor*, a multi-center study from 10 provinces of China revealed that work hours per week and number of patient for daily service were positively correlated with burnout score (both p < 0.001) The average work hours per week were 54.1 ± 10.7 and the average number of daily service patients was 27.8 ± 25.1, with doctors reported 60 or more work hours per week was independently associated with higher incidence of medical mistakes [20]. A multi-center study in Shanghai reported that burnout was higher among doctors who worked 40–60 h/week or over 60 h/week than those who worked up to 40 h/week (OR = 3.63, p < 0.001; OR = 4.54, p < 0.001) [19]. A multi-center study in Liaoning reported that 61.9% of respondents working > 40 h/week, and these doctors had significantly higher emotional exhaustion and cynicism scores and lower professional efficacy scores than respondents who worked fewer hours [21].

*Regarding to the work setting/environment factor*, a multi-center study in Shanghai reported that burnout was higher among doctors who worked on shift (OR = 2.85, p < 0.01). The study also suggested that hospital type closely correlates to job burnout. Burnout was higher among doctors who worked in hospitals of Grade II or Grade III than those who worked in Grade I hospitals (OR = 2.55, p < 0.001; OR = 3.62, p < 0.001) [19]. A multi-center study from 10 provinces of China revealed that the proportions of doctors with serious burnout were 10.9, 17.3, and 23.7% (p < 0.001) in primary, secondary, and tertiary hospitals respectively. Similarly, the burnout score was highest in tertiary hospitals (2.6 ± 1.1), followed by secondary (2.4 ± 1.1) and primary (2.0 ± 1.2) hospitals [20]. A multi-center cross-sectional survey from 10 areas in China also supported that doctors working in tertiary hospitals (F = 2.34, p = 0.04) or emergency department (p < 0.05) suffered from higher degree of burnout [33]. In general, the physical work environment was one of the major predictors for emotional exhaustion reported in a cross-sectional survey conducted in 2006. The study also revealed that surgeon and doctors working in the internal medicine wards scored significantly higher in job burnout than their colleagues (p < 0.05) [36]. The findings of higher burnout in surgeon and physician, and having physical work environment as significant predictor for emotional exhaustion were supported by another cross-sectional study conducted in 2008 [37]. A multi-center study in Liaoning revealed that 47.2% of doctors reported serious dissatisfaction with their relationship with patient; this was one of the most salient factors related to high emotional exhaustion, and cynicism and low professional efficacy scores [21].

*Regarding to the sociodemographic factor*, higher level of burnout was shown in doctors of younger age in three cross-sectional surveys: The prevalence of burnout cases was significantly higher among doctors under the age of 35 [19]. The score of exhaustion was significantly higher in the 30–40 year age group than that in any other groups in study conducted in 2008 [37]. This age group also scored highest in exhaustion in a study conducted in 2006 [36]. Higher level of burnout was also shown in doctors with less work experience in two cross-sectional surveys: The prevalence of burnout cases was significantly higher in those worked for less than 5 years [19]. The degree of burnout was higher among doctors under 10-year seniority (p = 0.01) [33]. The burnout prevalence was higher among doctors with a family income of less than 1000 CNY/month/person than among those with an income of more than 3000 CNY/month/person (OR = 5.63, p < 0.05) [19]. The burnout prevalence was higher among unmarried doctors compared with married doctors (OR = 1.76, p < 0.05) in a study conducted in Shanghai [19]. Similar findings of higher emotional exhaustion (t = 2.12, p = 0.03), higher depersonalization (t = 2.06, p = 0.04) and lower personal accomplishment (t = − 3.38, p = 0.00) were found in unmarried doctors when compared with married doctors in a survey conducted in 10 areas of China [33]. On the other hand, work-family conflict was associated with burnout among Chinese doctors in a cross-sectional study conducted in Liaoning [38].

*Regarding to the individual perception to work and life*, a cross-sectional study in Shanghai concluded that low job control, low reward and over-commitment were some of the most significant predictors of burnout [19]. Similar findings were reported in a study in Liaoning suggesting that variables that predicted burnout including high over-commitment, high psychological job demands, low reward, low decision authority, low supervisor and co-worker support [21]. Occupational stress was significantly positively related to all burnout dimensions (p < 0.05) in a study conducted in Henan. The study also noted that main significant predictors of burnout included role overload, role insufficiency, responsibility, and social support [37]. Another study in 2006 suggested that major predictors to burnout included role overload, role insufficiency, responsibility, social support, leisure activities and self-care [36]. A statistically significant negative correlation between organizational justice and job burnout (r = − 0.298, p = 0.000) was observed in a cross-sectional survey with 135 medical interns in Shanghai [39].

On the other hand, burnout was negatively associated with higher job satisfaction in a study among emergency doctors [22]. A study in Liaoning reported that Psychological Capital was a mediator between work-family conflict and burnout, suggesting that it might be a positive resource to reduce the negative effect of work-family conflict on burnout of doctors in China [38]. Coping resources were inversely correlated with the three dimensionalities of job burnout (p < 0.05) in the study conducted in Henan [37].

## Discussion

The result to research question one echoed with previous Western review studies [40, 41]: that burnout among doctors has reached epidemic levels, with overall prevalence of burnout symptoms among doctors in China ranged from 66.5% [19] to 87.8% [33].

The result to research question two suggested negative impact of burnout among doctors in China including association with anxiety symptoms and low job satisfaction at the individual doctors’ level, prone to committing medical mistakes affecting patient safety and higher turnover intention at the society/organizational level. The finding of these negative impacts also shared the same conclusions with previous Western studies.

However, this search reveals that studies focus on burnout among doctors in China is small in number when compared to the West, despite the significant prevalence and negative impacts of burnout among doctors in China. Interestingly, with long working hours, tremendous workload, deterioration of the overall doctor-patient relationship with one-third of doctors experienced conflict and thousands to have been injured [26], what are the reasons to enable this profession with high burnout and low morale continue to sustain the service delivery to the society? Burnout, which is a phenomenon raise from the West, has been translated in Chinese literature, with meaning closed to “fatigue” and “over-worked”. Chan [42] and Lo [43] first pointed out that existing Chinese translation may have limitation as they stressed more on the physical and under-describe the psychological and spiritual aspects of burnout. They introduced a new Chinese translation of burnout (Rán jié) which implies a 3-dimensional burnout, namely physical, psychological and spiritual levels [42, 43]. Hence, a deeper level of analysis regarding the cultural perspective is performed to explore the psychological, social and spiritual levels of burnout for Chinese doctors. The main discussion would focus on the reflection from the results to research question three as explored from the Chinese cultural perspective.

### Cultural reflection

In the present systematic literature review, most burnout literature mainly focused on reporting the alarming figures of prevalence and discussion on the adverse consequences of burnout. Factors contributing to doctor’s burnout were discussed in a generalized manner with minimal address to specific cultural consideration. Potential strategies to tackle burnout have been proposed in a generic way without tailor-made to doctors from different parts of the world. Literature in the present review has neither made any attempt to explore the burnout phenomenon from the cultural or moral perspectives, nor to account for the apparently high elasticity of Chinese doctors despite the adverse condition.

With specific reference to the Chinese context, ‘Chinese doctors’ consist of both qualities of “Chinese” and “doctors”. The Chinese attitude to working or fulfilling one’s duty is to stand all the hardship without complaining tiredness or fatigue until the end of his life. This is regarded as compliance to rites (*Li*) with moral respect. This inherited traditional working attitude may result in a self-expectation of maintaining a higher moral standard than the social norm, to stand all the hardship without complaining, hence prone to burnout. Although Chinese doctors can sense that they are overloaded or burnout, the Chinese culture of standing all the hardship without complaint may account for the high elasticity of this high burnout population and the sustainability of the medical service despite the adverse condition.

#### Shift of social culture and loss of humanity in medicine

In the past several decades, there is a world trend of diminishing humanity in medicine. China is no exception. Doctors are expected to be in pace with the rapid growing research findings, following clinical guidelines issued by international authorities in their daily practice. While the emergence of evidence-based guidelines and sub-specialization ensures highly standardized treatment, what people may have overlooked, is the impact of the over-emphasis on “specialization” on the existing doctor-patient relationship, which has a fundamental role affecting the functioning of any helping professional [44].

In the present review, the untrusting and distancing doctor-patient relationship is one of the salient factors for doctors’ burnout. In China, it is commonly found that patients may have doubts upon the general practitioners, or doctors practising in small-scale community hospitals with no specialization; the lack of relatively high technology was associated with less competence in medical treatment. This untrusting doctor-patient relationship has driven the patients to visit different specialists for problems of different organs in the body. This phenomenon explains our review findings that doctors working in tertiary hospitals in China are more overloaded and have higher burnout prevalence. This phenomenon, however, is uncommon in the Western world especially in Europe,^1^ despite the similar presence of high technology and sub-specialization in tertiary hospital. Strengthening primary health care has been a central health policy for years in Europe. The concept of having a holistic family doctor coordinating the health triage system has been well implanted, expressed in the health seeking behaviour. With a strong primary healthcare system, doctors working in the tertiary hospitals in the West are likely to be proportionally unloaded. Patients seek medical opinion from their own family doctor in their communities first, with regular and long-lasting doctor-patient relationship, in contrast to the one-off encounter between patient and a sub-specialist, with their relationship ceased once the relevant medical problem was resolved.

Another impact violating the traditional doctor-patient relationship in China is the shift of healthcare concept from delivering a medical mission to maintaining a medical business [44]. Shifting the doctor-patient relationship to business model is driving the doctors away from his mission of helping patients, relieving patients from sufferings with passion. In addition, medicine becomes a transaction between a “*medical service provider*” and a “*customer*” who pays for this service. The concept of “*customer*” has reinforced the patients’ beliefs that “whoever can afford a higher charge would get a higher quality medical service”. This “*medical service provider*-*customer relationship*” is clearly different from the “*traditional doctor*-*patient relationship*” which was well documented in ancient China medical textbooks such as the afore-mentioned “*On the Absolute Sincerity of Great Physicians*”. This changing relationship could be one of the root causes for the current negative perception to work and life, as one of our major finding addressed to research question three.

#### Impact of cultural shift and conflicting values on Chinese young doctors

One of the major findings in this review is that, young doctors in China, especially for those younger than 40 years old, have higher prevalence of burnout. Analyses have been raised in previous literatures, that it may be explained by the fact that, young doctors serving as trainees or junior posts in general are more overloaded, more likely to work more than 60 h/week, less rewarded with monthly wage less than $RMB 2000 and having less working experience. According to the first author’s frontline training experience, young doctors from different provinces in China do show disagreement and feel uncomfortable about the current “*medical service provider*-*customer relationship*” in general. One young doctor from Xi-an^2^ expresses desperate feeling when doctors are commonly reminded by the “*patient*” during the start of the medical consultation, that his family is very wealthy and demand for the best medical treatment. This implies patients have assumed doctor was of low moral standard and would only provide good medical service to wealthy people. The young doctor perceives himself as a “good” doctor with high moral duty and feels not being respected. Another young doctor from Shanghai^3^ sadly suggests that one way to manage angry patients in order to avoid complaint, is having no physical nor verbal defence even when being beaten or blamed by “*patients*”. She drops tears in the doctor-patient communication skill workshop when describing her way of managing angry patients, because she is very frustrated with the current low social status of Chinese doctors.

It comes to a point of psychological struggling, when young Chinese doctors primed with the Confucian Chinese culture, expecting high moral respect from the public initially, become very disappointed after discovering the reality of the current social environment. Before a new rationalisation to these young doctors is being formulated, they may be prone to burnout at the psychological and spiritual levels, with depression and anxiety symptoms; and may even responded by giving up the medical career, explaining the high turnover rate of medical graduates noted by the major research findings in this review study. Furthermore, young doctors belong to early to mid-adulthood of the life stages, somewhat have to accomplish their many responsibilities according to the society’s script, facing stressors like finding a home and mate, establishing a family or circle of friends, and/or getting a good job [45].

One may wonder how the Chinese doctors can stand for these tremendous hardships without complaining. In fact, the art of achieving a good balance between work and social life, especially in the life stage of young doctors, seems neither addressed in the Chinese culture nor in the medical education. Medical students are taught to become good doctors in medical schools. The definition of a “good” doctor that includes a high moral standard in traditional Chinese culture has been well documented and implanted into the heart of Chinese. The medical professionals have to bridge the traditional high moral standard and nowadays patients’ demands. To strive an art of work-life balance, is to formally include medical humanities in the medical curriculum in medical school in China and other parts of the world. This is what we advocated in the present review as the “renaissance of medical humanities”.

In this review study, unsatisfactory doctor-patient relationship was found to be one major predisposing factor for burnout among doctors in China. It may be too superficial to regard this as the root-cause, because this is only a sign of an underlying problem, i.e. loss of medical humanities. The original motivation driving a doctor to stand all the hardship, in order to relieve patients’ sufferings; humanity as the major reason of maintaining harmony between doctor and his patients, was well documented in ancient Chinese medical textbooks and historical record. Hence, by organizing workshops to enhance doctors’ communication skill can polish the tool of delivering medical service, but it is insufficient to target at the root-cause, i.e. loss of medical humanities.

Medical humanities may have partial overlapping to the art of medicine, but it is not identical. It comprises not only communication skills, professional judgement and illness management, but also the unmeasurable deeply immersed hearts of empathy, dual respect, genuineness, self-awareness and reflective practices. Medical humanities are not a technical skill that can be acquired by attending lectures and passing relevant examinations, it is a long process of the growth and maturation of a doctor, requires years of role-modelling and guided practice. While both undergraduate and postgraduate medical education reform by strengthening medical humanities in the curriculum is important, public education to achieve renaissance in medical humanities in the society cannot be neglected. If the social culture is skeptical of virtue, glorifies success by monetary measurement, disvalues altruism and self-actualization, it is impractical to expect our young doctors to maintain the mission of doctoring in an adverse social environment. While improving the remuneration package and working environment of doctors in China are very important, returning moral respect to doctors from the society is one of the most crucial needs for burnout prevention. Promotion of humanities may not be only beneficial to the medical profession and the sustainability of the healthcare system, but also to the genuine harmony of the Chinese society.

### Strategies of managing burnout among Chinese doctors

Western literature [40, 41] suggests that interventions for burnout can be classified into *doctor*-*individual level* and *organizational level*. The former usually involves mindfulness techniques or cognitive behavioural techniques to enhance job competence and improve communication skills, personal coping strategies, stress management and self-care training, etc. The later involves reduction of workload, changes in work schedule or more ambitious changes to the operation of practices and whole health care organizations, with improvement to teamwork, changes in work evaluation, supervision to reduce job demand, enhance job control, and increasing the level of participation in decision making [40]. A recent meta-analysis suggests that these recent intervention programs for burnout in doctors were associated with small benefits that may be boosted by adoption of organization-directed approaches, supporting that burnout is a problem of the whole health care organization, rather than individuals [41]. However, these findings and suggestions are not specific to Chinese doctors and without consideration to the cultural perspectives. Whether these interventions can be directly applied to the Chinese locality is subject to question. One of the major factors for low utilization and high dropout rates in Chinese mental health services, known as the stigmatization of mental illness, have long been recognized in previous literatures [46]. This is probably more significant among Chinese medical doctors, whose self-perception belongs to a higher moral standard and are thus more resilient to hardship when compared with majority of Chinese people. For instance, personal counselling or stress management workshops may have been shown by Western studies to be effective in burnout management, the Chinese work culture encouraging people to stand all the hardships without complaining may cause Chinese doctors unwilling to seek for help due to their compliance to *Li* and preserving to ‘face’. In order to overcome the barrier and facilitate the acceptance of counselling practice in the Chinese communities, professional counsellors should take into consideration of the cultural perspectives when they are counselling Chinese people especially when adopting Western theories and approaches [47–49].

The “Healthy China 2030” [50] health care reform policy which includes strengthening of primary health care, promotion of family doctors and disease prevention, can hopefully triage and reduce patients attending tertiary hospitals directly, hence decreasing the workload of doctors working in the tertiary hospital setting; promotion of medical humanities to both the medical discipline and the general population is crucial to combat burnout among doctors and maintain the sustainability of the medical system in a society built upon with Chinese culture.

### Limitation of the review

Although each of the 11 studies were multi-centred, had population of more than 100 respondents, and response rates > 75%, all of them belong to cross-sectional and exploratory studies which may have lower level of evidence when compared with such as Randomized Controlled Trials (RCTs) by using the evidence-based practice hierarchies. However, by applying a set of scientific criteria on qualitative studies exploring on a humanistic research topic can be limiting and may not be able to reflect the genuine picture. Another problem is that, although most studies used the MBI as burnout measurement tool, it is difficult to make direct comparisons between the various studies as different versions of the MBI were used, and information on the content, number of items, scoring methods and cut-off scores are varied. Doctors participated in these studies are also largely diversified from different specialities, work settings, and years of training, etc. with insufficient documentation in some of the studies. This affects the strength of the conclusions resulted from the review. Furthermore, although studies published in Chinese have also been included in the search process, some Chinese studies which do not have English abstracts may not be included in the Western databases used in this review process and probably be missed out.

### Future research direction

It is encouraging to see that most of the studies were carried out in the past 5 years, suggesting that burnout among doctors are getting more attention in the field and looking for a way out. It would be of interest to investigate whether East Asian countries (e.g. Korea, Taiwan, Japan) influenced by Confucian teaching would have similar cultural impact in their doctor burnout problem. While research on the cost-effectiveness of the respective healthcare reform measures to relieve burnout at the organizational level is important to refine health policy implementation, qualitative studies on the promotion of medical humanities to both the medical profession and the public are the way forward.

## Conclusion

This systematic review shows that burnout among doctors in China has reached epidemic level, with adverse consequences noted at both the individual doctors’ level and the society/organizational level, echoing with the findings in Western literature. Young doctors and doctors working in tertiary hospitals are more at risk of burnout, probably related to shift of social culture, loss of medical humanities and a weak primary healthcare system. Potential strategies of managing burnout in Chinese doctors therefore have to take consideration from the Chinese cultural perspective with renaissance of medical humanities and strengthening the primary healthcare system in China.

## Footnotes

1 The first author had a discussion with general practitioners from Denmark, Sweden, New Zealand and UK during a visit to a general practice clinic in Copenhagen, at the World Family Doctors’ Conference in 2016. The primary health care policy is very mature in Europe, with each citizen enrolled to the care of his own family doctor in the community with long-term relationship. The citizen cannot attend tertiary hospitals directly except in emergency. Hence, the overloading phenomenon for doctors working in tertiary hospitals is not commonly seen as what happening in China.

2 The first author was appointed as Honorary Professor of the First Affiliated Hospital of the Xi-an Medical School providing training on communication skill and medical humanities. During her stay in August 2016, the first author led a hot discussion with more than 100 local doctors in a communication skill workshop began by the question on why doctors felt frustrated when repeatedly reminded by patients that they were wealthy and could pay for the drug with the highest charge.

3 The first author conducted a workshop in June 2016 in Hong Kong, providing communication skill training to a group of doctors coming from different provinces of China. One of the case scenarios for demonstration was how to manage angry patients. Doctors from the floor were invited to share their ways of management in daily practices first, before the author shared her own experience with them.
